# The Effects of Combined Low Frequency Repetitive Transcranial Magnetic Stimulation and Motor Imagery on Upper Extremity Motor Recovery Following Stroke

**DOI:** 10.3389/fneur.2019.00096

**Published:** 2019-02-19

**Authors:** Wenxiu Pan, Pu Wang, Xiaohui Song, Xiaopei Sun, Qing Xie

**Affiliations:** ^1^Department of Rehabilitation Medicine, Ruijin Hospital, School of Medicine, Shanghai Jiao Tong University, Shanghai, China; ^2^Department of Rehabilitation Medicine, Shanghai Ruijin Rehabilitation Hospital, Shanghai, China

**Keywords:** stroke, upper limb, motor function, repetitive transcranial magnetic stimulation, motor imagery

## Abstract

**Objective:** To investigate the effects of low frequency transcranial magnetic stimulation (LF-rTMS) combined with motor imagery (MI) on upper limb motor function during stroke rehabilitation.

**Background:** Hemiplegic upper extremity activity obstacle is a common movement disorder after stroke. Compared with a single intervention, sequential protocol or combination of several techniques has been proven to be better for alleviating motor function disorder. Non-invasive neuromodulation techniques such as repetitive transcranial magnetic stimulation (rTMS) and motor imagery (MI) have been verified to augment the efficacy of rehabilitation.

**Methods:**Participants were randomly assigned to 2 intervention cohorts: (1) experimental group (rTMS+MI group) was applied at 1 Hz rTMS over the primary motor cortex of the contralesional hemisphere combined with audio-based MI; (2) control group (rTMS group) received the same therapeutic parameters of rTMS combined with audiotape-led relaxation. LF-rTMS protocol was conducted in 10 sessions over 2 weeks for 30 min. Functional measurements include Wolf Motor Function Test (WMFT), the Fugl-Meyer Assessment Upper Extremity (UE-FMA) subscore, the Box and Block Test (BBT), and the Modified Barthel index (MBI) were conducted at baseline, the second week (week 2) and the fourth week (week 4).

**Results:** All assessments of upper limb function improved in both groups at weeks 2 and 4. In particular, significant differences were observed between two groups at end-intervention and after intervention (*p* < 0.05). In these findings, we saw greater changes of WMFT (*p* < 0.01), UE-FMA (*p* < 0.01), BBT (*p* < 0.01), and MBI (*p* < 0.001) scores in the experimental group.

**Conclusions:** LF-rTMS combined with MI had a positive effect on motor function of upper limb and can be used for the rehabilitation of upper extremity motor recovery in stroke patients.

## Introduction

Decreased mobility of hemiplegic upper limb is a common dyskinesia after stroke. At present, clinical researchers have established a number of treatments to improve upper extremity motor function (1). Compared with a single intervention, a combination approach of different techniques has been proven to be better for alleviating movement disorder (2). Lots of trials have shown that movement function improvement after stroke can be enhanced by non-invasive brain stimulation techniques combined with conventional clinical practice (3–6).

Repetitive transcranial magnetic stimulation (rTMS) is one of non-invasive brain stimulations, and could modulate cortical activity. Stroke is considered to be one possible reason for imbalance of interhemispheric cortical inhibition. rTMS could rebulid the interhemisphere balance by down-regulating the excitability of the non-lesioned hemisphere with low frequency stimulation or up-regulating the lesioned excitability by high frequency stimulation (6). Randomized controlled trials have shown that short courses of inhibitory, contralesional rTMS can improve the motor function of hemiplegia after stroke (7, 8). Evidence suggested that maximum control of the lesioned hemisphere is associated with better function (9, 10). Early damage affected the ability of upper motor neurons to compete with lateral neurons to dominate motor neurons (11). Inhibition of contralateral primary motor cortex (M1) with 1 Hz rTMS may enhance hemispheric motor function. This method has revealed efficacy in the stroke rehabilitation for adults although they do not share the same models (8). Recently, the positive effects of HF-rTMS and LF-rTMS on movement disorder after stroke have been supported by accumulating evidence (7). And LF-rTMS has been confirmed to be in correlation with improved function in patients with chronic stroke (12, 13). Nowadays, a meta-analysis by Zhang et al. evaluated the therapeutic potential of LF-rTMS on stroke-induced upper limb movement disorder and cortex plasticity. This research supported that, as an add-on therapy, LF-rTMS successfully alleviated the hemiplegic upper limb motor deficit and significantly promoted upper limb function improvement after stroke (14).

Another non-invasive neuromodulation technique-motor imagery (MI), has been validated to increase the efficacy of rehabilitation and improve the performance of tasks associated with MI in patients after stroke (15–17). The functional recovery of most stroke patients occurred mainly in the first 3 months, and the functional gain obtained in the chronic phase was limited (18). A possible cause of limited functional recovery in the chronic phase is learned nouse. Patients with severe impairment cannot use their paretic limbs in daily activities may be the reason (19). MI is a dynamic state during which the subject mentally simulates a specific movement without any obvious movement (20). It means that MI has no strict restrictions on the patient's upper limb motor function, so it can be applied to stroke patients with poor function in chronic phase. According to previous studies, MI and motor execution share the same neural networks related to motor function (17, 21, 22). These findings support the idea that MI can be used as a substitute for physical exercise which is difficult for patients to do (23). MI training was assumed to enhance motor recovery in stroke rehabilitation (24). Based on traditional rehabilitation training, MI training is more effective than conventional training alone (17). For example, Kang et al. and Xu et al. demonstrated an increase in neural activity in the motor area during MI training (25, 26). And Kawakami et al. also investigated changes of cortex in reciprocal inhibition following MI in patients with chronic stroke, and reported positive plastic changes during mental practice with MI (27). In another pilot study, Mihara et al. demonstrated that NIRS-mediated neurofeedback MI could enhance the ipsilesional premotor area activation in correlation with MI training and could have significant effects on the motor deficit recovery in stroke patients. Besides these findings, they also found that the change of cortical activation was related to the recovery of the hand function (19).

In view of the fact that rTMS and MI have no strict restrictions on the limb function of patients with chronic stroke, this study intends to combine the two interventions to maximize the motor function recovery of patients. As the author know, few studies explore whether the effect of LF-rTMS can be enhanced by combining with MI on upper extremity activity. In this study, we hypothesize that combination therapy of LF-rTMS with MI training will promote recovery from upper limb movement disorder in patients after chronic stroke; we also predict that activities of daily living might improve accordingly.

Therefore, the objective was to investigate the effects of LF-rTMS combined with MI on improving motor functions of hemiplegic upper extremity in chronic stroke patients.

## Methods

### Participants

We recruited chronic patients with ischemic stroke from Shanghai Ruijin Rehabilitation Hospital over a 10 months period. The inclusion criteria as follows: (1) being diagnosed of ischemic stroke through neurological examination and CT or MRI scans for the first time; (2) inpatients within 3 to 12 months from the onset of diagnosis of ischemic stroke; (3) without upper-limb function impairments before this illness of stroke; (4) being aged 21 or over but no more than 80; and (5) Mini Mental State Examination score of 23 or more; (6) signed the consent form before the study start. The exclusion criteria were: (1) cerebral hemorrhage; (2) medically unstable such as severe liver and kidney malfunction, cardiopulmonary insufficiency, or malignant tumor; (3) aphasia, severe cognitive impairment, history of mental illness; (4) a history of epileptic seizures in the last 1 months or taking antiepileptic drugs recently; (5) pregnant; (6) severe visual and hearing impairment; (7) pacemaker, internal electrode, metal implants *in vivo*, and skull defects.

### Randomization

This study was a single-blinded randomized controlled trial; patients were given a number by a computer-generated randomization table when they were enrolled. Investigators who evaluated the outcomes were blinded from the group to which each patient was assigned. Patients did not know whether the audio-based MI delivered to them was specific or not. All participants who met the criteria were assigned to two groups according to their random number.

Participants were assigned to 2 intervention cohorts: (1) rTMS+MI group was applied at 1 Hz rTMS over the primary motor cortex of the contralesional hemisphere combined with audio-based MI; (2) rTMS group received same therapeutic parameters of LF-rTMS, and was applied audiotape-led relaxation.

**Figure 1:**
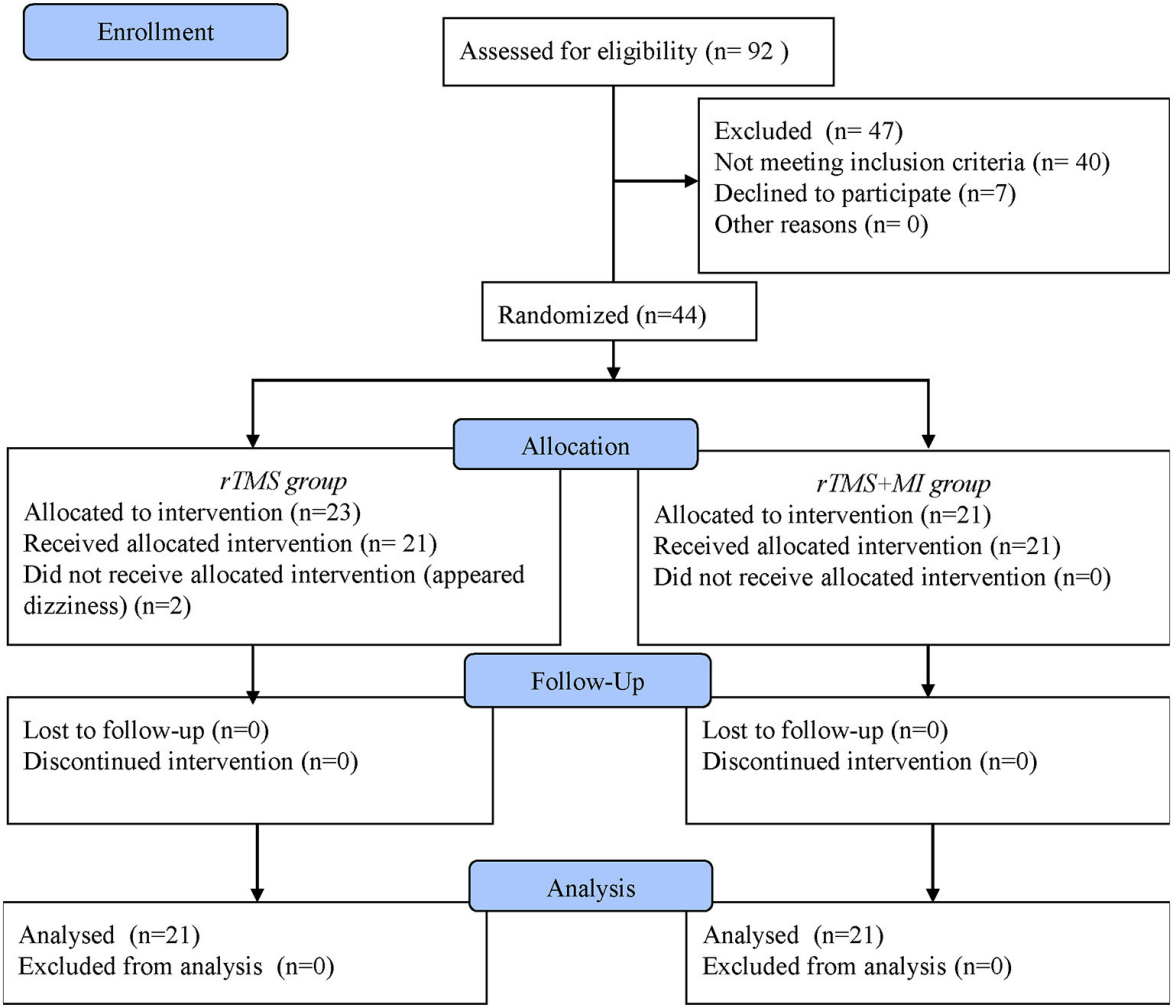
Flow Diagram of the Trial.

LF-rTMS was conducted in 10 sessions over 2 weeks for 30 min. Both groups received the same dosage of conventional rehabilitation on top of their interventions. Details of the flow diagram showed in Figure 1.

### Interventions

The proposal was approved by Shanghai Ruijin Rehabilitation Hospital Research Ethics Committee. The study was registered in the Chinese Clinical Trial Registry (ChiCTR) on http://www.chictr.org.cn/index.aspx. And the registration number is ChiCTR-INR-17012845. All patients participating in this study completed the consent form. The process of treatment didn't harm the participants. If there was any discomfort during the treatment, the patient had the right to stop the process at any time. We were assured that the patient had fully understood the purpose of the study and were aware of their rights, and then signed a written consent or fingerprint. Forty-two patients were randomized into two groups. On top of their interventions, all the participants received the same standardized conventional clinical rehabilitation involved physical therapy and occupational therapy for 120 min once a day for 10 sessions (5 d/w, 2 weeks), these programs included improving strength, posture, coordination, activities of daily living, etc., and mainly focusing on upper limb movements. But it's important to note that any specific training involved in mental practice with MI was not embedded in physical therapy.

#### LF-rTMS Pattern

A TMS stimulator with by a 90 mm eight-shaped coil and CCY-IV stimulator (Yi Ruide Company, Wuhan, China) was used. The participants were in supine position on the bed. And 1 Hz rTMS was applied to the unaffected hemisphere over the primary motor cortex. Each rTMS session consisted of 1,500 pulses, lasting 30 min. The stimulation site was defined as location where the largest motor evoked potential (MEP) in the first dorsal interosseous (FDI) muscle of the contralesional hemisphere was elicited upon surface electromyography (sEMG).

The resting motor threshold (RMT) of the FDI muscle of the contralesional upper extremity, by definition, was the minimum stimulus intensity produced the minimal motor evoked response of the muscle at rest (28, 29). And the amplitude of the MEP was about 50 μV produced in at least 5 of 10 trials (7, 8). Based on the measured RMT level, the stimulation intensity was set to 90% of the RMT of the FDI muscle. As the RMT may change during the whole study, we adjust the intensity for each session. Each patient received a total of 10 sessions over 2 weeks (5 d/w).

#### Audio-Based MI

Thirty minutes structured sessions were designed for audio-based MI training. All tasks were standard activities following a detailed audio-based instructions that not allowed to be individualized. The MI sessions developed in a quiet room in the hospital, and all the participants were in supine position on the bed. Structured details were presented as follows:

- The first 3 min of each session was reserved for preparation: leading participants to immerse themselves in the imaginary environment. As the audio-based instructions described, participants closed their eyes, relaxed their breathing, and gradually entered the imaginary state.
- The next 10 min of per session was reserved for warm up mental practice: let participants to imagine a variety of elementary joint relaxation activity for the upper limb of the affected side (such as raising of the arm, rotation of the wrist, opening and closing of the hand, etc.) (30, 31).
- A further 15 min per session was reserved for activities of daily living: such as writing a person's name on paper, pushing the door open, folding a piece of paper, drinking a bottle of water, open a book, open the door with a key, turn the light on and off, etc. (31).
- The final 2 min of each session was reserved for cooling down, leading participants to return to the real world from the imaginary environment (30).

#### Audiotaped-Led Relaxation

To ensure that each subject was as relaxed as possible during the mental practice with MI, soothing music was added to the audio-based instructions as the background. Considering that music may have a certain impact on the final results (32), we set up a audiotape-led relaxation program containing soothing music as a placebo-controlled group.

- In the first 3 min, subjects were asked to immerse themselves in imagination.
- During the next 25 min, the subjects continued to maintain their eyes closed and be relaxed.
- The final 2 min of each session were consistent with audio-based MI group.

There were two highlights of this program: the music was all about static objects such as buildings, animals, and landscape rather than sports; and there was no requirement for the participants' imaginary content.

In order to verify their involvement in mental practice, participants were asked open questions about the content of imaginary sensations throughout the sessions. For example, the researcher would ask whether the patient can imagine the specific situation or not and the clarity of the imagination according to the audiotaped-instructions. Furthermore, to ensure their MI ability, each patient underwent a the Kinesthetic and Visual Imagery Questionnaire (KVIQ-10) before the trial, and their imaginary questionnaire scores were all >25 points (33). MI training was applied at a total of 10 sessions for 2 weeks (5 d/w, 30 min/d).

After randomization, experimental group (rTMS+MI group) received 1 Hz rTMS over the unaffected upper limb of the primary motor area, and 30 min audio-guided mental practice with MI in the meantime. Control group (rTMS group) received 1 Hz rTMS over the primary motor area of the contralesional hemisphere, and 30 min audiotape-led relaxation simultaneously.

### Outcome Measurements

Information about patients' demographic characteristics and medical history were collected. All evaluations were conducted at baseline (the day before intervention), weeks 2 and 4. All participants were conducted by an independent evaluator who knew nothing about treatment or group's task allocation.

Primary outcome measure: The motor functions of the hemiplegic upper limb were measured by WMFT. Secondary outcome measures included: UE-FMA subscore; MBI, and BBT. The research procedure was approved by the Research ethics Committee of the Shanghai Ruijin Hospital. All participants completed and signed an informed written consent form before enrollment in this study.

### Statistical Analysis

We chose the last observation when participants dropped out, that means if the subject did not finish the study, missing values were replaced by the last evaluation score of the variable. Any statistical demographic and characteristics differences at baseline were explored with one-way ANOVAs. The statistical analysis was performed using SPSS 22.0. The Shapiro-Wilk test revealed that the data were normally distributed in our study, and the data also satisfied the test of homogeneity of variance. Independent sample *t*-test was used to evaluate differences between groups; paired sample *t*-test was used to test for differences between pre-and post-interventions in each group. Data were presented as means + standard deviation. *p* < 0.05 was considered statistically significant.

## Results

Subjects were randomly assigned to two groups and 42 patients (rTMS+MI group *n* = 21; rTMS group *n* = 21) had finished the trial. No participants dropped out during the process and in the follow-up. No participants reported severe side effects and no severe discomfort was reported in the whole study. There were no significant differences in the general characteristics of participants in the experimental and control groups (Table 1). Recovery between baseline and post-intervention were evident on all outcome variables (Table 2).

**Table 1:** Demographic and general characteristics of participants.

	**rTMS+MI group**	**rTMS group**	***P*-value**
	***n* = 21**	***n* = 21**	
**DEMOGRAPHICS**			
Age (year), mean ± SD	63.38 ± 6.45	64.14 ± 4.49	0.61
Gender-Male (%)	16 (76%)	12 (57%)	0.19
Onset (month), mean ± SD	4.96 ± 1.07	5.13 ± 1.09	0.30
**COMORBIDITIES**			
Hypertension *n*%	18 (86%)	17 (81%)	0.52
Diabetes *n*%	16 (76%)	15 (71%)	0.40
Atrial fibrillation *n*%	2 (9%)	4 (19%)	0.26
**PRIMARY OUTCOMES, MEAN** **±** **SD**			
WMFT	34.86 ± 6.68	34.43 ± 10.50	0.88
**SECONDARY OUTCOMES, MEAN** **±** **SD**			
UE-FMA	37.19 ± 5.78	35.86 ± 7.80	0.54
MBI	64.81 ± 5.51	63.00 ± 9.29	0.46
BBT	5.95 ± 3.70	6.05 ± 6.50	0.96

**Table 2:** Pre- and post-intervention of clinical outcome measures.

	**rTMS+MI group**	**rTMS group**	***P*-value**
	***n* = 21**	***n* = 21**	
**PRIMARY OUTCOMES, MEAN** **±** **SD**			
**WMFT**			
Baseline	34.86 ± 6.68	34.43 ± 10.50	0.879
Week 2	54.10 ± 8.16^###^^**^	43.62 ± 10.3^##^	0.001
Week 4	52.90 ± 9.57^###^^**^	42.42 ± 10.14^#^	0.002
**SECONDARY OUTCOMES, MEAN** **±** **SD**			
**UE-FMA**			
Baseline	37.19 ± 5.78	35.86 ± 7.80	0.542
Week 2	50.52 ± 6.00^###^^**^	43.33 ± 7.86^##^	0.002
Week 4	49.24 ± 6.52^###^^**^	42.14 ± 7.81^##^	0.003
**MBI**			
Baseline	64.81 ± 5.51	63.00 ± 9.29	0.458
Week 2	86.90 ± 5.61^###^^***^	72.57 ± 8.50^##^	0.000
Week 4	86.04 ± 6.33^###^^***^	72.24 ± 9.51^##^	0.000
**BBT**			
Baseline	5.95 ± 3.70	6.05 ± 6.50	0.955
Week 2	18.42 ± 6.21^###^^**^	12.05 ± 7.66^#^	0.006
Week 4	17.67 ± 6.31^###^^**^	11.43 ± 7.50^#^	0.007

# p < 0.05 (compared with baseline).

## < 0.01 (compared with baseline).

** p < 0.01 (compared with rTMS group).

### p < 0.001 (compared with baseline).

*** p < 0.001 (compared with rTMS group).

p-value (rTMS+MI group compared with rTMS group).

*WMFT, wolf motor function test; UE-FMA, Fugl-Meyer Assessment Upper Extremity; MBI, modified Barthel index; BBT: box and block test*.

### Wolf Motor Function Test

**Figure 2:**
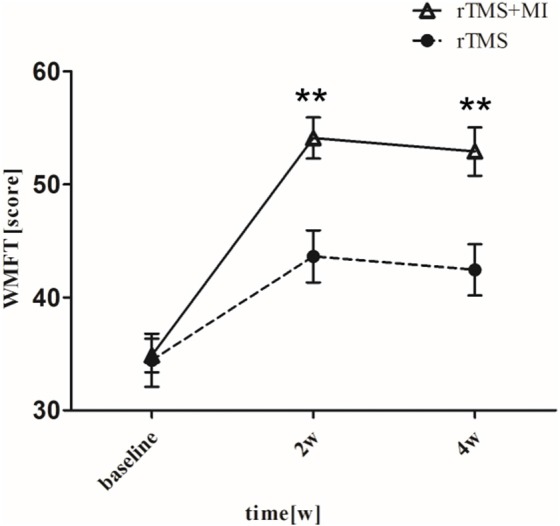
Pre- and post-intervention changes in Wolf Motor Function Test. ^**^p < 0.01 (compared with rTMS group).

There was a significant difference baseline and post-intervention in both groups (*p* < 0.05). In particular, significant differences were observed between two groups at weeks 2 and 4 (*p* < 0.01) (Figure 2).

### Fugl-Meyer Assessment Upper Extremity

**Figure 3:**
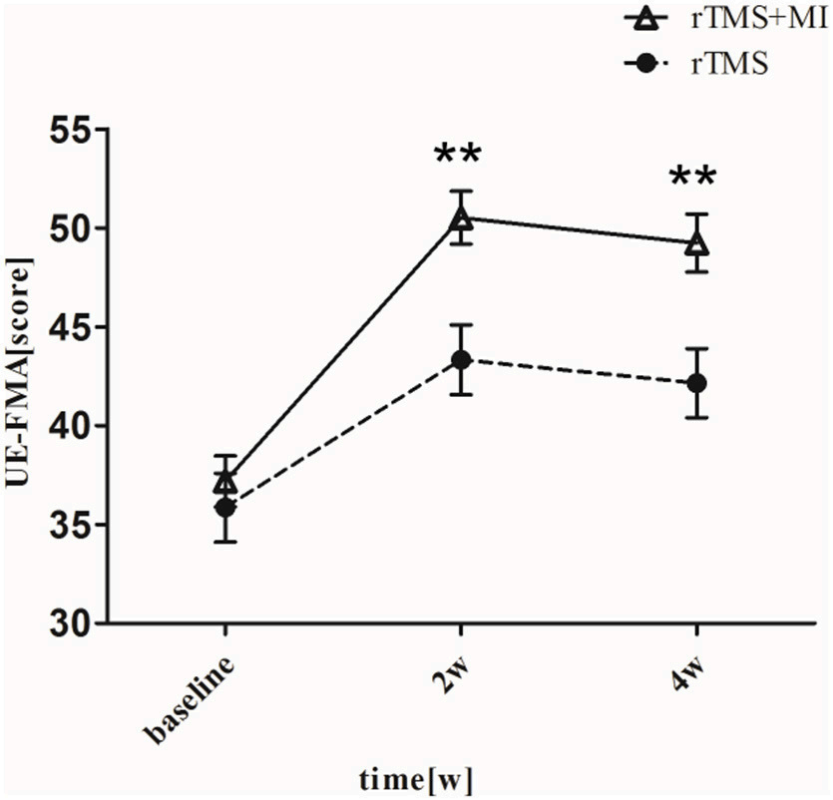
Pre- and post-intervention changes in Fugl-Meyer Motor Assessment Upper Extremity. ^**^p < 0.01 (compared with rTMS group).

UE-FMA increased from 37.19 ± 5.78 to 50.52 ± 6.00 (*p* < 0.001) in the rTMS+MI group, from 35.86 ± 7.80 to 43.33 ± 7.86 (*p* < 0.01) in the rTMS group at week 2. And comparison between the two groups suggested a significant difference at weeks 2 and 4 (*p* < 0.01) (Figure 3).

### Modified Barthel Index

**Figure 4:**
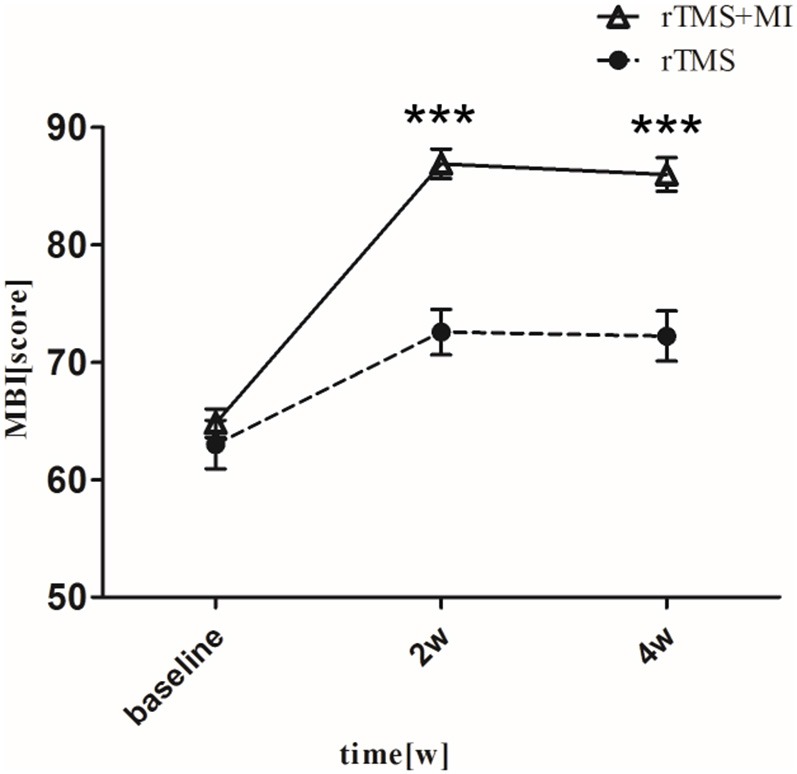
Pre- and post-intervention changes in Modified Barthel Index. ^***^p < 0.001 (compared with rTMS group).

With regard to the modified barthel index, a significant difference was observed in both groups at week 2 (*p* < 0.01). At week 4, MBI increased from 64.81 ± 5.51 to 86.04 ± 6.33 (*p* < 0.001) in the rTMS+MI group, from 63.00 ± 9.29 to 72.24 ± 9.51 (*p* < 0.01) in the rTMS group. Comparison of the differences between the groups revealed a significant difference at weeks 2 and 4 (*p* < 0.001) (Figure 4).

### Box and Block Test

**Figure 5:**
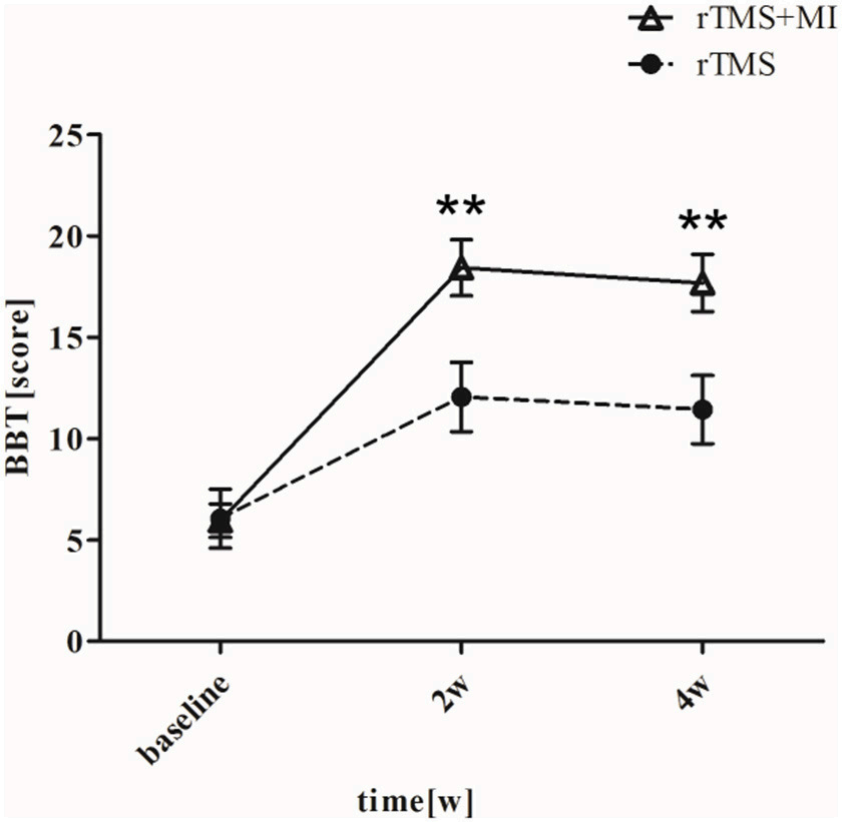
Pre- and post-intervention changes in Box and Block Test. ^**^p < 0.01 (compared with rTMS group).

BBT increased from 5.95 ± 3.70 to 18.42 ± 6.21 (*p* < 0.001) in the rTMS+MI group, from 6.05 ± 6.50 to 12.05 ± 7.66 (*p* < 0.05) in the rTMS group at week 2. Significant differences between the groups were found in the BBT at week 2 (*p* < 0.01) and week 4 (*p* < 0.01) (Figure 5).

## Discussion

As aforementioned, a combination approach of different techniques was better for improving movement function. For instance, Koyama et al. combined NMES with rTMS in patients with moderate to severe dysfunction after stroke, and they found that the combination protocol was more beneficial for recovery of stroke-induced motor function compared with the control group (34). Zheng et al. (35) made an investigation that whether the combination of 1 Hz rTMS and virtual reality (VR) could facilitate the motor function recovery of upper extremity in patients after stroke. After intervention, they reported a better improvement in upper limb motor function in LF-rTMS and VR group compared with the control group who underwent sham LF-rTMS of the unaffected hemisphere (35).

Since a combination of several rehabilitation techniques could be better for improving the motor function in stroke patients, then we investigated whether the combination 1 Hz rTMS and mental practice with MI could alleviate the motor function of the affected upper limb in patients with stroke. Because numerous studies have reported the positive effects of LF-rTMS for promoting motor recovery following stroke compared with clinical rehabilitation, we did not design a conventional practice group as control. The results showed that all functional assessments of upper limb improved, and patient's daily living ability improved accordingly after the interventions. In addition, compared with rTMS group, greater changes of WMFT (*p* < 0.01), UE-FMA (*p* < 0.01), BBT (*p* < 0.01), and MBI (*p* < 0.001) scores in the rTMS+MI group were showed in this study. And these findings were similar to many researches: Mihara et al. showed significant improvements in UE-FMA, and Zheng et al. suggested improvements in WMFT (19, 35). More meaningful is that our study addressed the hypothesis that a combination of LF-rTMS and MI training had a positive effect on motor function of upper limb and can be used for the rehabilitation of upper extremity motor recovery in chronic stroke patients.

Clinical studies have found that patients with stroke have altered their collaborative working ability of multiple brain regions, such as decreased connectivity between the premotor area and the primary motor area, increased inhibition of the affected hemisphere, and functional connectivity of these abnormalities are significantly correlated with the degree of motor function decline (36–38). As we known, rTMS could regulate the magnitude of transcallosal inhibition and influence the information interaction of brain functional regions (14, 39). Studies have shown that the activation of the ipsilateral primary motor area (M1) is associated with motor function recovery and amelioration (40, 41). In Jin et al. study (42), the effects of rTMS combined with motor training on brain neural activities were investigated based on the method of brain network. Electroencephalography in resting state with eyes closed was recorded before and after rTMS combined with motor training. They found that the changes of functional connectivity could be detected mainly between functional regions rather than inside regions. The functional connectivity at lower frequency band (theta and alpha) was significantly increased, and was opposite at higher frequency band (beta, gamma1, and gamma2). Furthermore, they found that the rTMS combined with motor training had a significant influence on the functional connectivity between central region in non-dominant hemisphere and dominant frontal regions and non-dominant frontal regions at alpha frequency. The sensorimotor cortex (SMC) region, especially the primary motor area (M1) of the anterior central gyrus, is closely related to the motor ability (27, 43, 44). After 4 weeks of mental practice with MI, Sun Limin et al. revealed two kinds of brain remodeling modes: recruitment activation and concentrated activation of the ipsilateral SMC region. With the recovery of upper limb motor function, there was a tendency to activate the SMC on the affected hemisphere. Therefore, the entire motor system saw a substantial increase in the efficiency of information output owing to the increasing activation of contralateral sensorimotor cortex (cSMC) (30).

In our findings, greater improvements in functional assessments of upper limb was showed in the experimental group. There are several possible reasons: compared with audiotaped-led relaxation, MI training may enhance the sensory information input; and repeated imaginary training accelerated the formation of normal motor reflex arc, thereby improving neuromuscular function. We assumed that, after 14 days of rTMS combined with MI, the abnormal functional connection between the brain regions may be changed to some extent. In addition, because of the improvement of motor cognitive ability, complicated motion could be completed with only low efficiency.

There are also several limitations in our study: this is a single-center trial, and the current sample size is not sufficient for subgroup analysis of covariates such as location or sides of stroke infarction. And limited follow-up time, does not provide us with information on long-term rehabilitation effects. Besides, we did not have a conventional clinical practice group as control, the absence of a conventional rehabilitation group is a pity of this study. Another limitation is that we did not perform neuroimaging studies to describe the consistent results with clinical scores. In the future, with the help of visualization technology MI, we will make it more accurately and provide further evidence including functional magnetic resonance (MR) or neuro-electrophysiological technique to demonstrate plastic changes in the brain following the intervention. In conclusion, further studies including a larger number of subjects with long-term follow-up assessments are needed.

## Conclusion

Inspite of the abovementioned limitations, this research proves that the combination of LF-rTMS with MI may be a positive method for improving motor function of upper limb in patients after chronic stroke.

## Ethics Statement

This study was carried out in accordance with the recommendations of Evidence-based guidelines on the therapeutic use of repetitive transcranial magnetic stimulation (rTMS), Shanghai Ruijin Rehabilitation Hospital Research Ethics Committee with written informed consent from all subjects. All subjects gave written informed consent in accordance with the Declaration of Helsinki. The protocol was approved by the Shanghai Ruijin Rehabilitation Hospital Research Ethics Committee.

## Author Contributions

QX and PW designed and supervised the study. XhS and XpS performed the patient selection and training. WP evaluated the data and wrote the manuscript.

### Conflict of Interest Statement

The authors declare that the research was conducted in the absence of any commercial or financial relationships that could be construed as a potential conflict of interest.
